# Response: “Newborn chicks need no number tricks. Commentary: Number-space mapping in the newborn chick resembles humans' mental number line”

**DOI:** 10.3389/fnhum.2016.00031

**Published:** 2016-02-05

**Authors:** Rosa Rugani, Giorgio Vallortigara, Konstantinos Priftis, Lucia Regolin

**Affiliations:** ^1^Center for Mind/Brain Sciences, University of TrentoRovereto, Italy; ^2^Department of General Psychology, University of PadovaPadova, Italy

**Keywords:** numerical cognition, number sense, domestic chick, spatial numerical associations, mental number line

Newborn chicks do need number (magnitude) tricks, exactly like humans. Shaki and Fischer (2015) have argued that our results, according to which newborn chicks associate smaller numbers with the left space and larger numbers with the right space (Rugani et al., 2015), could be due to non-numerical cues such as area, perimeter, or density of the displayed dots. Regarding to our Experiment 3, they claimed “…*chicks in all control conditions responded to both numerosity and at least one uncontrolled dimension, as they were in the first and second experiments, too*.” In Experiment 3 (Rugani et al., 2015) we actually performed three separate conditions to control for non-numerical cues. In the first condition, we controlled for shape, size, and color of each element. In the second condition we controlled for the overall area (summation of the areas of all elements depicted in each stimulus), and occupancy (the overall space occupied by each group of elements). In the third condition, we controlled for the overall perimeter (summation of the perimeters of all elements depicted in each stimulus), density (the mean distance among the elements), and occupancy. Notably, in the third condition a negative correlation was there between overall area and number: the overall area of the 32 elements was smaller than that of the 20 elements (training), and the overall area of the 20 elements was smaller than that of the 8 elements. Had chicks associated space to overall area, instead of number, their choices would have been the opposite to what we found. Thus, the association small numbers/left space and large numbers/right space holds even when the possible use of non-numerical cues is prevented, falsifying Shaki and Fischer's hypothesis.

Furthermore, chicks' behavior in the third condition explains why the supposed role of the light is unfounded. Shaki and Fischer (2015) stated that “*There is an established association…of light with left…this “left-light bias” may explain why chicks turn to the left in the presence of fewer dots compared to their training*.” However, given that in one of our control condition there was a negative correlation between overall area and number (with perimeter and density held constant), smaller numbers were associated with larger (not smaller) areas and, therefore, with *less* light. Again chicks behaved the opposite to what predicted by Shaki and Fischer. Furthermore, the figure reported in Shaki and Fischer's comment is misleading. Indeed, this is a figure depicting the small versus large number test of our Experiment 1, and not one of the conditions, in which we controlled for the quantitative cues in Experiment 3.

Shaki and Fischer also suggested that the complexity, and not the number, of the patterns could explain the association with space: “*less and more complex patterns are associated with left and right space (Beaumont*, *1985**; Heath et al*., *2005**)*.” We regard the comment as non-pertinent to our context, as these papers investigated aesthetic preferences in adult humans in front of stimuli depicting different figures. Beaumont (1985) asked participants to arrange two different pictures. Participants positioned the most complex figure on the left of the less complex one. We did not ask chicks to perform anything at all like that. Furthermore, the two stimuli employed in each test trial with chicks were identical: none could be considered neither more, nor less, “complex” than the other.

Heath et al. (2005) asked participants (Roman script readers [English], Arabic script readers, Biliterates, and Illiterates) to express their aesthetic preference by indicating one of two, vertically arranged, mirror compositions. Each composition was made by three different, horizontally arranged elements. Results showed different complex patterns of aesthetic preference, as a function of reading direction. We did not use this kind of stimuli with our chicks (i.e., on each trial we used two identical stimuli). Moreover, we did not ask our (illiterate) chicks to perform aesthetic judgments (i.e., we did not regard chicks' spontaneous choices as aesthetic judgments). Therefore, the “complexity” criticism by Shaki and Fischer is not founded.

Overall our results, in particular in the control conditions, are best explained by a bias that induces an association of smaller numbers with the left side and larger numbers with the right side. The main argument of Shaki and Fischer is that when dealing with non-symbolic numbers there are, unavoidably, continuous physical variables (i.e., sensory cues) that co-vary with number. This is certainly true. However, Approximate Number System (ANS) researchers do in fact think that numerosity estimates are computed from sensory cues. Indeed, different sensory properties combine to a common abstract (non-sensory) representation of number (or better, as we should point out below, of *magnitudes*). We believe that here resides our theoretical disagreement with Shaki and Fischer. They seem to believe that dimensions like duration, distance, location, brightness, and so on *are not* numbers. In contrast we believe, following Gallistel (2011), that they are indeed all numbers, in the sense that numbers are *symbols for magnitudes*. The SNARC effect is in fact so ubiquitous and multimodal, precisely because it deals with magnitude (quantity); magnitude includes numbers, but is not limited to it. Shaki and Fischer seem to attribute a particular status to numbers as representations of discrete quantity (numerosity). We believe, instead, that numbers are a part of a more general system for representing quantity, whether discrete or continuous. Gallistel (2011) has argued eloquently why it is necessarily to be so. Many continuous quantities are derived by an arithmetic combination of a discrete and a continuous quantity (e.g., rate). Therefore, the brain must possess a system to represent both discrete and continuous quantities within the same symbolic currency.

## Author contributions

RR and GV wrote the manuscript. LR and KP provided critical revision. All authors approved the final version of the manuscript for submission.

### Conflict of interest statement

The authors declare that the research was conducted in the absence of any commercial or financial relationships that could be construed as a potential conflict of interest.
